# Associations between objective measures of physical activity, sleep and stress levels among preschool children

**DOI:** 10.1186/s12887-020-02108-7

**Published:** 2020-05-27

**Authors:** Dagny Y. Eythorsdottir, Peder Frederiksen, Sofus C. Larsen, Nanna J. Olsen, Berit L. Heitmann

**Affiliations:** 1 Research Unit for Dietary Studies, The Parker Institute, Bispebjerg and Frederiksberg Hospital, The Capital Region, Frederiksberg, Denmark; 2 Section for General Practice, Department of Public health, University of Copenhagen, Copenhagen, Denmark

## Abstract

**Background:**

Cortisol is often used as a biological marker for stress. When measured in urine or serum, representing a short-term measurement of the hormone, it has been associated with unfavorable sleep characteristics and both low and high physical activity levels. However, cortisol in hair represents a long-term stress measure and has been suggested as a promising new marker for chronic stress. Therefore, we aimed to examine the association between objectively measured sleep, physical activity and hair cortisol levels in preschool children.

**Methods:**

In order to obtain objective measures of physical activity and sleep habits, 54 children aged 2–6 years wore an ActiGraph for 5 consecutive days and nights. For chronic stress measurements of each child, hair was cut from the back of the head close to the scalp for analysis of cortisol levels. Associations between measured sleep quality and quantity and level of physical activity and hair cortisol levels were estimated using linear regression analysis, presented as β. Results were adjusted for sex, age and BMI z-score.

**Results:**

We found no significant association between log-transformed cortisol (pg/mg) and sleep duration (hours) (β = − 0.0016, *p* = 0.99), sleep efficiency (β = − 3.1, *p* = 0.18), sleep latency (β = 0.015, *p* = 0.16) or physical activity level (100 counts per min) (β = 0.014, *p* = 0.22). However, sleep latency (min) was directly associated with physical activity (counts per min) levels (β = 35.2, *p* = 0.02), while sleep duration (hours) (β = − 142.1, *p* = 0.55) and sleep efficiency (%) (β = − 4087, *p* = 0.26) showed no significant associations.

**Conclusions:**

In our study, a high physical activity level was associated with poorer sleep habits. Neither sleep quality nor physical activity were related to long term cortisol exposure. These results are among the first to study associations between objectively measured sleep, physical activity and chronic cortisol levels among preschool children. More and larger studies are therefore needed.

## Background

Cortisol is secreted from the hypothalamic-pituitary-adrenal (HPA) axis in diurnal cycles, which peaks shortly after waking and drops throughout the day. As cortisol is the end product that signifies the activation of the neuroendocrine system in response to stress and low blood-glucose concentrations in humans, it is frequently used as a measurement of stress among both children and adults [1]. Previous studies have reported associations between cortisol levels and sleep difficulties as well as physical activity (PA) in both adults [2–5] and children [6–10].

There are multiple ways of measuring cortisol, which include saliva, urine, blood and, more recently, hair. The latter measures long term cortisol levels (chronic stress) [11] while the three former are short term measurements (acute stress) [12]. Previous studies have suggested that cortisol in hair seems to provide a non-invasive measurement of long-term activity in the HPA-axis, and that long-term cortisol can be used as an indicator of both sub-acute and chronic stress [13].

Although little is known about the potential long-term health effects of elevated hair cortisol in children, a study from 2015 conducted in Sweden found that children with higher infant cortisol levels were significantly more affected by 12 of the 14 most common childhood diseases [14].

Cortisol measured in hair is a relatively new measurement, and therefore, to our knowledge, no previous studies have examined its relationship with sleep quality or PA in children. Previous studies that have examined the association between sleep quality and salivary cortisol levels among children seem to generally agree that shorter sleep duration and/or longer sleep onset latency, were directly related to cortisol measured in saliva, in both longitudinal and cross-sectional studies. For instance, two studies that examined the association of salivary cortisol on sleep characteristics among children (aged 18–20 months to 5 ½ years) both reported a higher morning cortisol among children that presented with poor sleep habits compared to those with better sleep habits [6, 15]. Similarly, two other studies, with the age ranges of 12–36 months and 6–10 years, which examined the association between sleep patterns and salivary cortisol levels, showed that awakening salivary cortisol was higher among those that had slept badly the preceding night [7, 16]. Additionally, one study reported, that after controlling for demographic variables, a higher afternoon salivary cortisol was related to self-reported sleep problems, including shorter sleep duration and poorer sleep quality among 7 to 11 year old children [8]. However, associations between sleep and cortisol, measured in saliva, serum or urine, are often modest or lacking [11, 17–19].

The association between PA and cortisol levels is still controversial. Only three studies were identified that examined the relation between PA and cortisol levels among children. Two of these studies reported that high PA was associated with higher salivary cortisol, measured directly after the PA was performed [9, 20], while Hershberger et al., who looked at salivary cortisol following a bout of exercise in both lean and obese children, found a tendency that post-exercise cortisol levels were lower among obese compared to lean subjects [10].

Thus, the purposes of the present study were to examine associations between objectively measured sleep quality and quantity and level of PA measured by Actigraphy and chronic stress measured by hair cortisol levels among pre-school children.

## Methods

Data from the current study was obtained from the Healthy Start Project (ClinicalTrials.gov, ID: NCT01583335) which was conducted between 2009 and 2011.

### Enrolment

The Healthy Start Intervention project aimed at preventing overweight and obesity in children aged 2–6 years, who were predisposed to overweight due to having either a high birth weight (> 4000 g), a mother who was overweight prior to pregnancy (body mass index (BMI) > 28), or a mother with low educational level (≤ 10 years) (subgroup only). Information on birth weight and pre-pregnancy BMI of the mother was obtained from the Danish National Birth Register, on all children born between 2004 and 2007 from selected municipalities in the Copenhagen area. Information on maternal educational level was obtained from administrative birth forms. A detailed description of the Healthy Start study has been published elsewhere [21].

All children eligible for inclusion were randomized into an intervention group, a control group, and a shadow control group. Only data from the intervention group and the control group was analyzed in the current study, as the shadow control group was followed via registers only and therefore did not provide information on the variables used in the present study. The intervention consisted of individual guidance in optimizing diet and PA habits, reducing chronic stress, improving sleep quality and quantity as well as participation in cooking classes and play arrangements. A total of 635 children from the intervention and control group participated in the baseline examination. Children who were overweight according to international criteria [22] were excluded from further study (*n* = 92).

Halfway through the intervention period a subgroup of 79 children from the intervention group, who were willing to wear an ActiGraph, participated in a sub-study where sleep and PA was measured. In this subgroup, we had some valid information on at total of 77 individuals [information on sleep (*n* = 68), PA (*n* = 54) and hair cortisol (*n* = 72)].

### ActiGraph GT3X

ActiGraph measures were obtained over a continuous period of 5 days and nights using ActiGraph GT3X. ActiGraph registers a person’s movements triaxially through an accelerometer on one single axis or multiple axes. The device catches movements between 0.25 Hz – 2.3 Hz, as previous studies have found that voluntary movements take place within this range [23]. The parents were instructed that if the child did not want to wear the device all 5 days, the most important time to wear it was during the night. Since a number of children only wore the ActiGraph during the night, only 54 observations were available for PA analysis while 68 children had information on sleep. The device was placed on the left wrist (right wrist if the child was left-handed) using a broad elastic band. If the elastic was too big for the wrist, it was placed on the upper arm or the ankle (left ankle if the child was right-handed and vice versa). The device was set to an epoch length of 60 s with normal filter level. Daily average PA as well as sleep over the 5 consecutive days was calculated, providing detailed objective information on the children’s PA and sleep habits. As evidence suggests that pre-school children exhibit low levels of moderate to vigorous PA (MVPA), high levels of inactivity [24] and lack the proficiency in motor skills which underpin more sophisticated activities such as sport [25], the overall activity level was obtained in counts per minute and not MVPA. Counts per minute were calculated from three different axes. The sleep variables that were identified from the ActiGraphs were: sleep latency (total time falling asleep, reported in minutes), sleep duration (time from the child fell asleep until it woke up, reported in hours) and sleep efficiency (the percentage of time from when the child fell asleep until final wake up, which was spent asleep). The program Actilife (ActiGraph, Pensacola, FL, USA), software version 5.0, with the algorithm developed by Sadeh et al. [26] was used for analyzing data from the ActiGraphs, both for PA and sleep analyses.

### Cortisol measurements

Stress/mental health was one of the domains the Healthy Start intervention was focused on, and measurements of hair cortisol were collected to provide a subjective stress measurement. Hair was sampled to obtain an objective measure of chronic stress. Information on frequency of hair washes and whether the hair was currently colored was also obtained in order to adjust for potential dilution of the hair cortisol level. However, subsequent analyses did not provide evidence to support that hair cortisol concentration is influenced by hair dyeing status or hair washing frequency [27]. The concentration of cortisol in hair samples, given as pg/mg hair, was determined by a modification of a previously described protocol [28]. Hair samples were cut from the posterior vertex as close to the scalp as possible. The hair sample was stored in aluminum foil, and the scalp end of the sample was carefully marked. Between 10 and 20 mg of hair from the 1–2 cm closest to the scalp was accurately weighed and minced finely with scissors. One milliliter of methanol was added and the suspension was incubated overnight at 50 °C with a gentle shaking. The following day, the methanol was transferred into a clean tube and evaporated to dryness under nitrogen. The residue was reconstituted in 250 μl PBS buffer (pH 8.0). The cortisol concentration in the resulting buffer solution was determined in duplicate using a commercially available salivary cortisol enzyme-linked immunosorbent assay (ALPCO Diagnostics, Salem, NH, USA). For the 996 hair samples collected from children and parents participating in the Healthy Start study, twenty-seven assays were conducted with an 8.0% intra-assay coefficient of variation. Each assay had a capacity of 40 hair samples and family members were analysed using the same assay. The assay sensitivity was 16.7 pg/mg based on a hair mass of 15 mg. The reproducibility of the assay determined by analysis of aliquots of the same hair samples in different assays was 15% [29].

### Statistical analysis

We had information on a minimum of 54 individuals, which gave approximately 85% power to detect correlations of 0.4 or greater absolute values. Cortisol measurements, which were non-normally distributed, were log transformed in order to make them normally distributed before analysis. The associations between log transformed cortisol and sleep latency, sleep duration, sleep efficiency and PA were estimated in separate models using linear regression. First, crude analyses were conducted. Secondly, sex, age and BMI z-scores were added to the models. Likewise, the associations between PA and sleep latency, sleep duration and sleep efficiency were estimated using linear regression and following the same adjustment scheme.

Normality of continuous variables and model assumptions (investigating linearity of effects on outcomes, consistency with a normal distribution and variance homogeneity) were assessed through visual inspection of histograms and residual plots.

A significance level of 5% was used. Stata 13.1 was used for all statistical analysis (StataCorp LP, College Station, Texas, USA; www.stata.com).

## Results

Information on age, sleep characteristics, hair cortisol levels as well as PA levels for the included children, stratified by gender are shown in Table 1. No differences in age, sleep patterns or PA levels were observed between boys and girls (all *p* ≥ 0.05).

**Table 1:** Characteristics for the included participants (*n* = 77)

	n	Overall Median (range)	n	Boys Median (range)	n	Girls Median (range)	*p*-value §
Age (years)	77	5.6 (3.1–7.3)	48	5.7 (3.1–7.3)	29	5.3 (3.5–6.9)	0.12
Sleep latency (min)	68	13.8 (0.0–57.5)§	44	13.3 (0.0–57.5)	24	14.0 (0.0–38.0)	0,90
Sleep duration (hours)	68	8.7 (6.3–11.3)§	44	8.7 (6.5–11.3)§	24	8.7 (6.3–10.2)§	0.50
Sleep efficiency (%)	68	0.81 (0.69–0-94)	44	0.81 (0.69–0.94)	24	0.81 (0.74–0.92)	0.49
Cortisol levels (pg/mg)	72	109 (7–890)	45	116 (7–890)	27	93 (8–291)	0.21
PA levels (CPM)	54	3080 (342–5282)	35	3293 (341–5282)	19	2483 (563–4158)	0.06

*Abbreviations:* PA: Physical activity; min: minutes; CPM: Counts per minute

§*P*-value for gender difference (Wilcoxon rank-sum test)

### Association between cortisol, sleep and PA

Regression analyses were performed for the relation between the three different sleep characteristics (sleep latency, −duration and –efficiency) as well as PA levels and cortisol levels. Sleep characteristics and PA were generally not associated with cortisol levels (all *p* ≥ 0.05). Adjusting the results for gender, age and BMI z-scores did not alter the results (Table 2).

**Table 2:** Association between log transformed cortisol (pg/mg) and sleep latency, sleep duration, sleep efficiency and physical activity before and after adjusting for sex, age and BMI z - scores

	Crude				Adjusted			
	R^2^	Β	95% CI	*p* value	R^2^	β	95% CI	*p* value
Sleep latency (min)	0.038	0.015	(−0.00, 0.035)	0.13	0.188	0.0084	(−0.011, 0.028)	0.40
Sleep duration (hours)	< 0.001	−0.0016	(−0.34, 0.34)	0.99	0.179	0.055	(−0.27, 0.38)	0.74
Sleep efficiency (%)^a^	0.030	−3.1	(−7.7, 1.5)	0.18	0.179	−0.60	(−5.3, 4.1)	0.80
Physical activity (100 CPM)^b^	0.033	0.014	(−0.008, 0.036)	0.22	0.178	0.013	(−0.009, 0.035)	0.22

^a^: percentage of minutes scored as sleep during the down time interval; ^b^ Counts per minute

### Association between sleep and PA

**Fig. 1:**
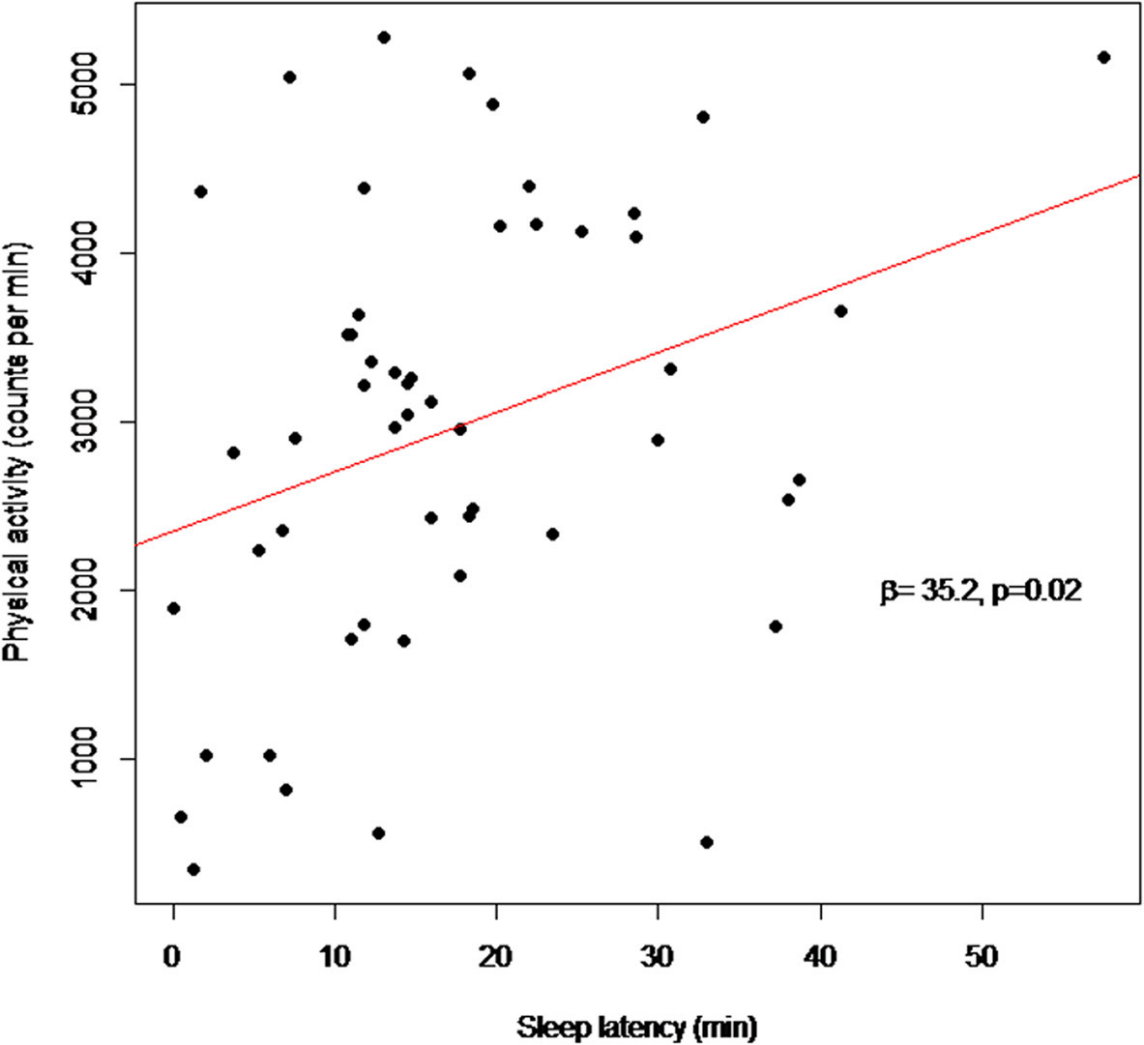
Associations between physical activity levels (counts per minute) and sleep latency (min)

A longer sleep latency (mins) was associated with a higher PA level (counts per minute) (β = 35.2, p = 0.02) (Fig. 1). PA was not associated with sleep duration or sleep efficiency (both *p* ≥ 0.05) and adjusting for covariates gave essentially similar results (Table 3).

**Table 3:** Association between physical activity (counts per minute) and sleep latency, sleep duration and sleep efficiency before and after adjusting for sex, age and BMI z - scores

	Crude				Adjusted			
	R^2^	β	95% CI	*p* value	R^2^	β	95% CI	*p* value
Sleep latency (min)	0.103	35.21	(5.67, 64.75)	0.02	0.152	33.65	(2.02, 65.28)	0.04
Sleep duration (Hours)	0.007	−142.1	(− 622.3, 338,1)	0.55	0.053	− 132.3	(− 620.6, 356,1)	0.59
Sleep efficiency (%)^a^	0.025	−4087	(−11,291, 3117)	0.26	0.071	− 4106	(− 11,948, 3736)	0.30

^a^: percentage of minutes scored as sleep during the down time interval

## Discussion

In the present study, we examined associations between objectively measured sleep and PA and chronic stress among preschool children. A direct association was observed between PA and sleep latency, but not between PA and sleep duration or sleep efficiency. No association was observed between sleep or PA and cortisol levels.

Results from previous studies in children around the same age have reported direct associations between poor sleep habits and cortisol levels from blood or saliva measures, representing current acute stress levels [6–8, 15, 16]. Similarly, a few earlier studies have found associations between PA levels and cortisol levels measured by blood or saliva [9, 10]. However, we were unable to identify previous studies that examined associations between sleep quality or PA and hair cortisol levels. Previous research has suggested that hair cortisol is only weakly associated with serum and saliva cortisol [12], and this may explain the lack of association between sleep quality and hair cortisol observed in our study. Furthermore, we had a relatively small sample size, and it is possible that we did not have the necessary statistical power to detect weak associations. Thus, confirmation of our results in future, preferably larger, studies are needed. Ideally, such studies should include measurements of both PA, sleep and long-term cortisol collected repeatedly over a longer period.

Our results suggest that it may take active 2–6-year-old children longer to fall asleep compared to children that are less active. These results are opposed to some previous studies that have showed a decrease in sleep latency among active children compared to those who were less active [30], while others only showed an inverse association if the PA was performed in the evening [31]. However, studies have primarily examined sleep characteristics among small groups of good sleepers, potentially with limited room for improvement [32]. Furthermore, few previous studies included children of similar ages as our study population [33]. Therefore, future long-term studies with more detailed information on objectively measured PA that examine if high PA, may influence sleep characteristics in this age group are still needed.

The present study has several strengths, primarily that all variables were measured rather than self-reported, thereby eliminating reporting bias. Other studies have examined the validity of the ActiGraph for measuring PA, compared to VO_2_ exhaustion, and showed that ActiGraph measures are valid for measuring both sleep [26] and PA [34] in children, and further suggesting a parental bias in the provided sleep diaries.

Furthermore, by measuring chronic levels of cortisol, we remove the diurnal effect on cortisol levels that acute measurements such as saliva and serum, are effected by [13]. To our knowledge, this is the first study to measure and examine both PA and sleep and cortisol levels among children.

However, our study also has some limitations, for instance the relatively low number of participants and hence low power to identify associations. Also, although we adjusted for several potential confounders, we cannot rule out that unmeasured or residual confounding influenced our results. Furthermore, since our study was cross-sectional in design, we cannot eliminate that cortisol levels influenced sleep or PA levels, rather than vice versa. By default, we placed the ActiGraph on the wrist of the child’s non-dominant hand. However, in a few cases where the elastic band was too big for the wrist, the device was placed on the upper arm or the ankle. Since we used algorithms developed for the wrist, this may have produced some cases of biased PA and sleep estimates and may also have contributed to the findings of weak associations as this bias may have attenuated some of the observed associations. Our results are also dependent on the assumption that the PA measured in the children represents their habitual PA, which is not necessarily the case. Additionally, some [12] but not all [19] previous studies have found a correlation between saliva or urine, and hair cortisol measurements. It is therefore quite possible that even if sleep and PA may truly associate with acute cortisol levels (measured in blood, saliva or urine), this may not necessarily be the case for chronic cortisol levels (measured in hair).

Finally, as the sub-group of 2–6-year-old children examined in the present study were all normal weight, susceptible to future obesity and participated in an intervention aimed at preventing future obesity. Hence, our results may not be generalizable to all children in a similar age group.

## Conclusions

Objectively measured sleep characteristics and activity patterns were not associated with chronic stress measured by hair cortisol levels among normal weight children aged 2–6 years, while a high PA may be related to longer sleep latency among this age group. However, more studies including larger samples of children and with objective measures of cortisol, sleep and PA over a longer period, are needed.

## Data Availability

In order to protect participant data, all data has been deposited at The Danish National Archives and is available upon request through http://dda.dk/simple-search, Archive number: 22248, search title: “Prevention of weight gain among normal weight, high risk, pre-school children - a randomized controlled interventions study, 2008”.

## Abbreviations

BMI: Body Mass Index
CPM: Counts per minute,
PA: Physical activity
